# Crystal structure of human CRM1, covalently modified by 2-mercaptoethanol on Cys528, in complex with RanGTP

**DOI:** 10.1107/S2053230X2100203X

**Published:** 2021-03-03

**Authors:** Alaa Shaikhqasem, Kerstin Schmitt, Oliver Valerius, Ralf Ficner

**Affiliations:** a Department for Molecular Structural Biology, Georg-August-Universität Göttingen, Justus-von-Liebig Weg 11, 37077 Göttingen, Germany; b Department of Molecular Microbiology and Genetics, Georg-August-Universität Göttingen, Grisebachstrasse 8, 37077 Göttingen, Germany

**Keywords:** nuclear export, cancer, exportin 1, cysteine modification

## Abstract

CRM1 is a nuclear export receptor that has been intensively targeted over the last decade for the development of antitumor and antiviral drugs. Structural analysis of several inhibitor compounds bound to CRM1 revealed that their mechanism of action relies on the covalent modification of a critical cysteine residue (Cys528 in the human receptor) located in the nuclear export signal-binding cleft. This study presents the crystal structure of human CRM1, covalently modified by 2-mercaptoethanol on Cys528, in complex with RanGTP at 2.58 Å resolution. The results demonstrate that buffer components can interfere with the characterization of cysteine-dependent inhibitor compounds.

## Abstract 2

The covalent modification of human CRM1 by 2-mercaptoethanol interferes with the characterization of cysteine-dependent inhibitor compounds.

## 1. Introduction   

CRM1 (chromosomal region maintenance 1) is an essential nucleocytoplasmic transport receptor that mediates the nuclear export of a wide range of proteins and ribonucleoprotein complexes (Güttler & Görlich, 2011 ▸; Kırlı *et al.*, 2015 ▸). CRM1-mediated transport is dependent on the cooperative binding of the small GTPase Ran in its GTP-bound form (RanGTP) and of the cargo protein to the export receptor, forming a trimeric complex that traverses the nuclear pore complex. The overall architecture of CRM1 consists of a ring-like structure that is composed of 21 HEAT repeats, each consisting of two antiparallel α-helices, A and B, connected *via* a short linker loop (Monecke *et al.*, 2009 ▸; Dong *et al.*, 2009 ▸). CRM1 recognizes cargo proteins by their leucine-rich nuclear export signal (NES) peptide. The NES peptide contains four or five hydrophobic residues that bind specifically into five hydrophobic pockets (Φ0–Φ4) located in the cleft between HEAT repeats 11A and 12A (referred to as the NES-binding cleft; la Cour *et al.*, 2004 ▸; Fung *et al.*, 2017 ▸). Overexpression of CRM1 has been observed in several cancers, and it has been identified as the major nuclear exporter for several onco­proteins, growth regulators and suppressor proteins such as p53, p21, BRCA1/2, Rb and FOXO, which lead to the initiation and progression of cancer (Hill *et al.*, 2014 ▸; Faustino *et al.*, 2007 ▸; Turner *et al.*, 2012 ▸; Watt & Leaner, 2010 ▸). Furthermore, CRM1 plays a key role in several viral diseases as it is co-opted by viruses such as influenza, rabies virus P and HIV for the nuclear export of their RNA and ribonucleoprotein (RNP) complexes. This renders CRM1 an interesting drug target for therapeutic intervention in several cancers and viral diseases (Mathew & Ghildyal, 2017 ▸; Dickmanns *et al.*, 2015 ▸).

Leptomycin B (LMB), a natural compound that consists of a polyketide chain with an α,β-unsaturated lactone ring, was the first CRM1 inhibitor to be discovered (Hamamoto *et al.*, 1985 ▸; Kudo *et al.*, 1998 ▸). Clinical tests revealed severe side effects and high toxicity (Newlands *et al.*, 1996 ▸), which induced a continuous search for and the development of alternative natural and synthetic compounds that could be used in CRM1 inhibition (Tamura *et al.*, 2010 ▸; Kau *et al.*, 2003 ▸; Liu *et al.*, 2015 ▸; Daelemans *et al.*, 2002 ▸; Bonazzi *et al.*, 2010 ▸; Sakakibara *et al.*, 2011 ▸; Mathew & Ghildyal, 2017 ▸). Structural analysis by means of X-ray crystallography defined the molecular basis of CRM1 inhibition. Furthermore, it has been used as a reliable approach for the development of novel CRM1 inhibitors (Kalid *et al.*, 2012 ▸; Sun *et al.*, 2013 ▸; Tian *et al.*, 2020 ▸). The crystallographic analysis of several natural and synthetic compounds bound to CRM1 defined a general mechanism of inhibition by the covalent modification of a reactive cysteine residue located in the NES-binding cleft of human CRM1 (Cys528). The binding of inhibitor compounds, mediated by the reactive cysteine, interferes with the binding of the NES peptide and prevents the formation of a stable export complex. As the human protein failed to crystallize in complex with inhibitors, CMR1 from the yeast *Saccharomyces cere­visiae* (*^Sc^*CRM1) was genetically modified to incorporate the reactive cysteine (T539C) and was used to crystallize CRM1 in complex with several inhibitor compounds (Lapalombella *et al.*, 2012 ▸; Etchin *et al.*, 2013 ▸; Haines *et al.*, 2015 ▸; Hing *et al.*, 2016 ▸; Tian *et al.*, 2020 ▸). Recently, we developed a crystallization approach using a stabilized variant of human CRM1 (*^Hs^*CRM1), with which we succeeded in solving the crystal structure of LMB bound to *^Hs^*CRM1 in complex with RanGTP (Shaikhqasem *et al.*, 2020 ▸). We furthermore investigated the molecular mechanism of several novel CRM1 inhibitors, including the compounds C3 {IUPAC name 2-[[1,1,1,3,3,3-hexafluoro-2-[(2-fluorobenzoyl)amino]propan-2-yl]amino]-4,5,6,7-tetrahydro-1-benzothiophene-3-carboxamide}, C6 {IUPAC name 4-(4-chlorophenyl)sulfonyl-*N*,*N*-dimethyl-2-(3,4,5-tri­methoxyphenyl)-1,3-oxazol-5-amine} and C10 {IUPAC name 5-methoxythieno[3,2-d][1,2]thiazole} (Fetz *et al.*, 2009 ▸; Shaikhqasem *et al.*, 2020 ▸). This investigation revealed that these three compounds disrupt CRM1–NES interaction due to their binding to *^Hs^*CRM1 at different rates in a Cys528-dependent manner (Shaikhqasem *et al.*, 2020 ▸). As the compound C6 exhibited the highest binding affinity among the tested compounds, we tried to crystallize it in complex with *^Hs^*CRM1 following the same approach as used for LMB. However, crystallographic analysis revealed an unexpected modification of the reactive cysteine in several data sets collected from different crystals of potential *^Hs^*CRM1–C6 complexes. Here, we present structural insight into the covalent modification of Cys528 of *^Hs^*CRM1 by 2-mercaptoethanol (BME), which was introduced as a buffer component during protein purification.

## 2. Materials and methods   

### 2.1. Macromolecule production   

A HEAT9 loop mutant (^430^VLV^432^ to AAA) of C-terminally truncated (α-helix; Δ1037–1071) *^Hs^*CRM1 (*^Hs^*CRM1^Δ^) was expressed and purified as described previously (Shaikhqasem *et al.*, 2020 ▸). The purification buffer in the last purification step contained 6 m*M* BME. Human RanGTP^1–180,Q69L^ (Monecke *et al.*, 2009 ▸) was prepared as described in Port *et al.* (2015 ▸). Macromolecule-production information for both CRM1 and Ran is summarized in Table 1 ▸.

### 2.2. Crystallization   

For crystallization, the complex was prepared following the same protocol as used to prepare the *^Hs^*CRM1^Δ^–RanGTP–LMB complex (Shaikhqasem *et al.*, 2020 ▸) with the exception of the addition of C6 (synthesized by ChemBridge Corporation, USA) in a ten-molar excess to CRM1 to counter its lower binding affinity in comparison with LMB. Single crystals with moderate diffraction quality grew within 3–6 days in the commercial crystallization buffer Morpheus H10 (Gorrec, 2009 ▸; Table 2 ▸) when mixed in a 1:1 ratio with the complex concentrated to 3 mg ml^−1^. Crystallization information is summarized in Table 2 ▸.

### 2.3. Data collection and processing   

X-ray diffraction data were collected on EMBL beamline P13 at PETRA III, DESY, Hamburg, Germany equipped with a PILATUS 6M detector. The collected data were indexed, processed and scaled using the *XDS* package (Kabsch, 2010 ▸). The data set revealed an orthorhombic lattice, with unit-cell parameters *a* = 121.11, *b* = 150.59, *c* = 231.97 Å, belonging to space group *I*222 (Table 3 ▸).

### 2.4. Structure solution and refinement   

The structure was solved by molecular replacement with *Phaser* (McCoy *et al.*, 2007 ▸) using the crystal structure of the *^Hs^*CRM1^Δ^–RanGTP–LMB complex (PDB entry 6tvo; Shaikhqasem *et al.*, 2020 ▸) as the search model. The structure was refined to reasonable *R* factors (Table 4 ▸) by iterative cycles of refinement and manual rebuilding in *REFMAC*5 (Murshudov *et al.*, 2011 ▸) and *Coot* (Emsley *et al.*, 2010 ▸), respectively. Water molecules were added with consideration of hydrogen-bond restraints and only if both *mF*_o_ − *DF*_c_ and 2*mF*_o_ − *DF*_c_ electron-density map peaks were simultaneously present at levels of 3.0σ and greater than 1.0σ, respectively. BME was modeled manually in *Coot*. A polder OMIT map (Liebschner *et al.*, 2017 ▸), generated by omitting both BME and Cys528, verified the presence of the Cys–BME conjugate. The root-mean-square deviation (r.m.s.d.) measurements were calculated using *LSQMAN* (Kleywegt, 1999 ▸). Figures were generated with *PyMOL* (version 1.8; Schrödinger).

### 2.5. Liquid chromatography–mass spectrometry analysis   

Four samples were prepared for liquid chromatography–mass spectrometry (LC-MS): *^Hs^*CRM1^Δ^, *^Hs^*CRM1^Δ^ mixed with crystallization buffer (Morpheus H10) and incubated overnight at 4°C to mimic the crystallization environment, crystals grown using the *^Hs^*CRM1^Δ^–RanGTP–C6 complex and crystals used for diffraction experiments. Cysteine-reactive chemicals, reducing agents and cysteine-modifying steps were avoided throughout the entire procedure. Equal volumes of the protein solutions were mixed with 2× SDS sample buffer (62.5 m*M* Tris–HCl pH 6.8, 2.5% SDS, 0.002% bromophenol blue, 10% glycerol). Crystals not exposed to X-ray radiation were transferred and dissolved in a drop containing 1× SDS sample buffer, while crystals used in diffraction experiments were carefully thawed in a drop of water and were then mixed with an equal volume of 2× SDS sample buffer. Afterwards, samples were boiled at 95°C for 5 min and applied onto an SDS–PAGE gel for brief separation. Protein-containing bands were cut and subjected to trypsin (SERVA Electrophoresis, catalogue No. 37283.01) digestion according to Shevchenko *et al.* (1996 ▸). Desalting of tryptic peptides prior to LC-MS was performed via StageTips according to the protocol described by Rappsilber *et al.* (2007 ▸). 2 µl of each sample was subjected to reverse-phase liquid chromatography for peptide separation using an RSLCnano Ultimate 3000 system (Thermo Fisher Scientific). The peptides were loaded onto an Acclaim PepMap 100 pre-column (100 µm × 2 cm, C18, 5 µm, 100 Å; Thermo Fisher Scientific) with 0.07% trifluoroacetic acid at a flow rate of 20 µl min^−1^ for 3 min. Analytical separation of the peptides was performed on an Acclaim PepMap RSLC column (75 µm × 50 cm, C18, 2 µm, 100 Å; Thermo Fisher Scientific) at a flow rate of 300 nl min^−1^. The solvent composition was gradually changed over 94 min from 96% solvent *A* (0.1% formic acid) and 4% solvent *B* (80% acetonitrile, 0.1% formic acid) to 10% solvent *B* within 2 min, to 30% solvent *B* within the next 58 min, to 45% solvent *B* within the following 22 min and to 90% solvent *B* within the last 12 min of the gradient. All solvents and acids were of Optima grade for LC-MS (Thermo Fisher Scientific). Eluting peptides were ionized online by nano-electrospray using a Nanospray Flex Ion Source (Thermo Scientific) at 1.5 kV (liquid junction) and transferred into a Q Exactive HF mass spectrometer (Thermo Fisher Scientific). Full scans in the mass range 300–1650 *m*/*z* were recorded at a resolution of 30 000 followed by data-dependent top 10 HCD fragmentation at a resolution of 15 000 (dynamic exclusion enabled). LC-MS method programming and data acquisition were performed with the *XCalibur* 4.0 software (Thermo Fisher Scientific).

MS/MS2 data were searched against an *Escherichia coli* specific protein database (UniProt Proteome ID UP000000625) that additionally contained the CRM1 sequence using *MaxQuant* 1.6.0.16 (Cox & Mann, 2008 ▸). The digestion mode was trypsin/P, and the maximum number of missed cleavage sites was set to two. Oxidation at methionine and N-terminal protein acetylation were set as variable modifications. A search for dependent peptides was performed to identify additional peptide modifications. The mass tolerances of precursors and fragment ions were 4.5 p.p.m. and 20 p.p.m. (HCD), respectively. False-discovery rates were calculated using the revert decoy mode, and the threshold for peptide-sequence matches as well as protein identifications was 0.01. *MaxQuant* output data were further evaluated using *Perseus* 1.6.0.7 (Tyanova *et al.*, 2016 ▸). The dependent-peptide search provided evidence for the presence of a DeStreak (2-mercaptoethanol; BME) modification (Δmass of 75.9983) at the cysteine residue of the CRM1 peptide DLLGLCEQK (Asp523–Lys531). Based on this result, the data were searched against the same database as before using *Proteome Discoverer* 2.2.0.388 with the *SequestHT* search algorithm and the DeStreak modification at cysteines as a variable modification. Precursor mass tolerance and fragment mass tolerances were 10 p.p.m. and 0.02 Da, respectively. The digestion mode and false-discovery rate were the same as for the *MaxQuant* analysis.

## 3. Results   

### 3.1. Identification of the modification of Cys528 by 2-mercaptoethanol   

**Figure 1:**
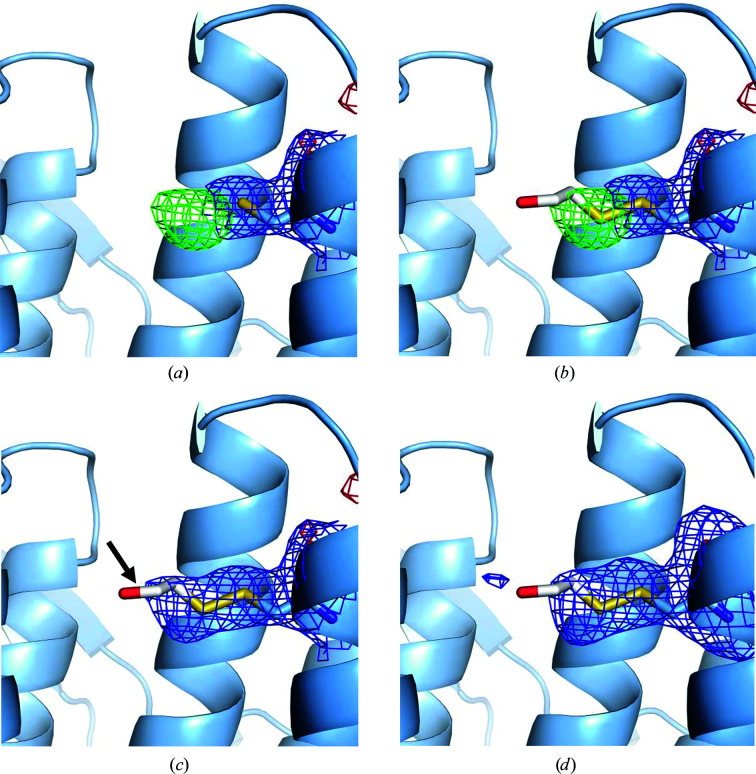
The modification of Cys528 by 2-mercaptoethanol (BME). 2*mF*_o_ − *DF*_c_ (contoured at 1.0σ in blue) and *mF*_o_ − *DF*_c_ (contoured at 3.0σ in green and −3.0σ in red) difference electron-density maps around Cys528 generated after model refinement with unmodified cysteine in (*a*) and (*b*) and after refinement of the covalently modified Cys528–BME conjugate model in (*c*). Nonmodified cysteine demonstrates an excess electron-density map representing a pronounced positive peak in the *mF*_o_ − *DF*_c_ map (*a*). The S atom of the modeled BME occupies the center of the excess electron-density map peak observed close to Cys528 (*b*). (*c*) Crystallographic refinement of the atomic model containing the Cys528–MBE conjugate. Neither positive nor negative peaks in the *mF*_o_ − *DF*_c_ map could be observed at contour levels of 3.0σ and −3.0σ, respectively. The methyl hydroxy moiety (indicated by an arrow) was not visible in the electron-density map due to its flexibility (rotational freedom). (*d*) A polder OMIT map of the Cys528–BME conjugate (contoured at 2.2σ in blue) confirms that Cys528 modification by BME explains the excess electron-density peak.

The main aim of the performed crystallization experiment was to gain structural insight into the interaction of C6 with CRM1. Our recent results showed that the compound exhibits a reduced inhibitory activity when Cys528 is changed to a serine, suggesting that the compound is binding to or is in the vicinity of the reactive cysteine in the NES-binding cleft (Shaikhqasem *et al.*, 2020 ▸). Structure refinement of several data sets obtained from crystals grown using the *^Hs^*CRM1^Δ^–*^Hs^*RanGTP–C6 complex displayed excess difference electron density at Cys528 (Fig. 1 ▸ *a*). Nevertheless, the positive density in the *mF*_o_ − *DF*_c_ map (3σ level) was much smaller in size than expected for the compound C6 and could not be explained by the compound. The center of the *mF*_o_ − *DF*_c_ electron-density peak (3σ level) was located within a distance of 2 Å from the S atom of Cys528, indicating a possible covalent modification. In order to confirm the type of modification and to elucidate whether it was introduced prior to crystallization or by synchrotron radiation, LC-MS analysis was performed for samples prepared from purified *^Hs^*CRM1^Δ^, the *^Hs^*CRM1^Δ^–RanGTP–C6 complex mixed with crystallization buffer (Morpheus H10), complex crystals that were not used for diffraction experiments and crystals exposed to synchrotron radiation.

**Figure 2:**
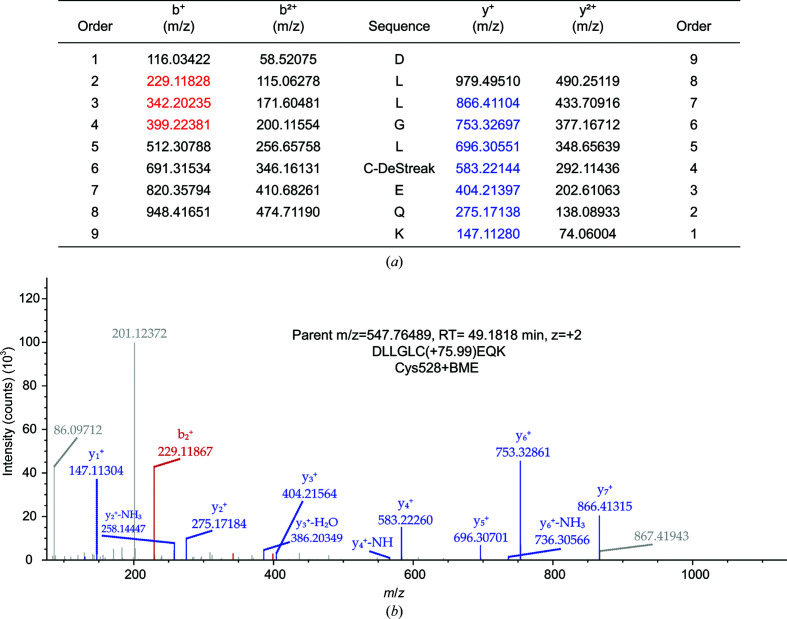
Determination of the Cys528 DeStreak modification of human CRM1 with 2-mercaptoethanol (BME) using mass-spectrometric analysis. (*a*) List of possible fragment ions of the Cys528–BME-containing peptide [DLLGLC(+75.99)EQK] with detected y ions in blue and b ions in red. (*b*) Representative fragmentation spectrum (1 of 15) of the respective precursor ion with an *m*/*z* of 547.765. RT, retention time.

The results of the LC-MS analysis revealed a mass difference of 75.9983 Da at the cysteine residue within the tryptic peptide DLLGLCEQK (the reactive cysteine Cys528; Fig. 2 ▸). The observed shift corresponds to a Cys–BME conjugate, revealing the covalent modification of Cys528 by BME, known as a DeStreak modification (Kim *et al.*, 2015 ▸). Although BME was introduced into the CRM1 buffer as a reducing agent during protein purification, the complex mixed with the crystallization buffer and the crystals of the complex before and after exposure to synchrotron radiation were more BME-bound compared with the purified protein. This further confirms that the reactivity of BME towards the cysteine is induced by the crystallization buffer conditions and that the observed excess electron-density map is not related to radiation damage caused by synchrotron radiation.

Crystallographic refinement of the atomic model containing the Cys528–BME conjugate explained the excess electron density (Figs. 1 ▸ *b* and 1 ▸ *c*), which was also further supported by a polder OMIT map calculated with *Phenix* (Liebschner *et al.*, 2019 ▸; Fig. 1 ▸ *d*). While the disulfide bond fits the electron-density map, the freely rotatable methyl hydroxy moiety was not visible in the density, indicating that it is most likely to be disordered.

### 3.2. Crystal structure of *^Hs^*CRM1 covalently modified by BME in the NES-binding cleft   

**Figure 3:**
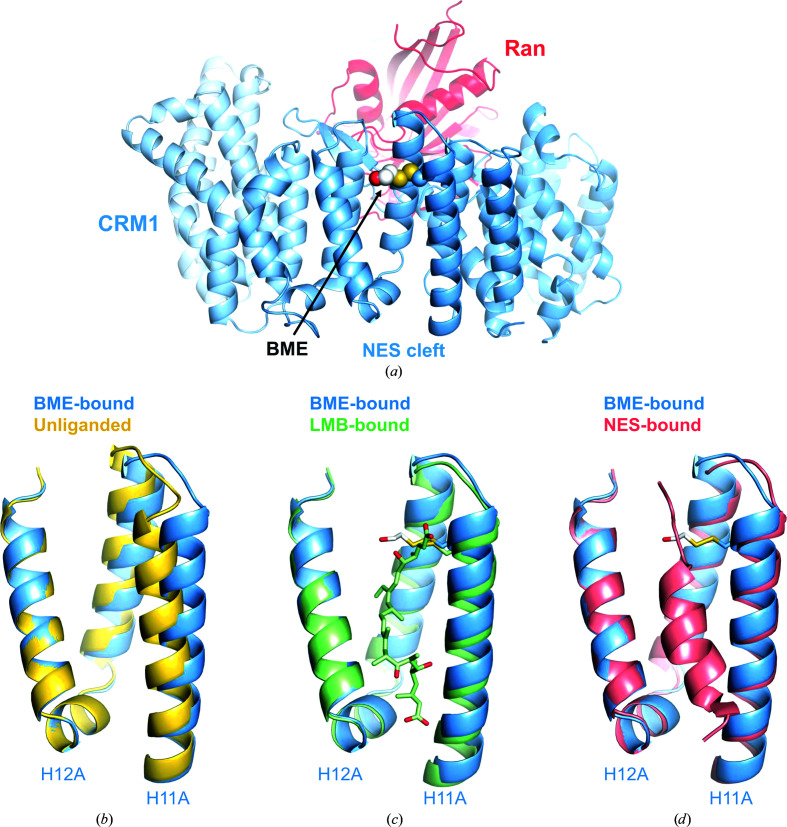
(*a*) Overall structure of the *^Hs^*CRM1–*^Hs^*RanGTP–BME complex depicted in cartoon representation. CRM1 is shown in light blue and Ran is colored red. The BME–Cys528 covalent adduct, located in the NES-binding cleft, is represented as spheres. Structural alignment of the BME-bound cleft with the unliganded cleft (yellow; PDB entry 3m1i; Koyama & Matsuura, 2010 ▸) is shown in (*b*), with LMB-bound CRM1 (pale green; PDB entry 6tvo; Shaikhqasem *et al.*, 2020 ▸) in (*c*) and with NES-bound CRM1 (salmon; PDB entry 3nby; Güttler *et al.*, 2010 ▸) in (*d*). BME is not shown in (*b*) and is depicted as sticks in (*c*) and in (*d*). LMB is depicted as sticks (pale green) in (*c*).

The structure presented here was obtained using the previously established protocol employed for crystallization of the *^Hs^*CRM1^Δ^–RanGTP–LMB complex. Similar to the structure of the LMB complex, the CRM1 molecule demonstrates an overall toroid-like compact conformation that results from the typical HEAT-repeat tandem arrangement (Fig. 3 ▸ *a*). Ran is bound in the inner core of the CRM1 toroid and its binding is stabilized by the acidic loop, which is arranged in a seatbelt-like structure. The NES-binding cleft, with BME covalently bound to Cys528, is in an open conformation when compared with the unliganded cleft of *^Sc^*CRM1 in complex with Ran and RanBP1 (C^α^ r.m.s.d. of 1.545 Å for the cleft residues 509–576 and the corresponding residues of *^Sc^*CRM1; Fig. 3 ▸ *b*). However, the BME-bound cleft exhibits a slightly narrower conformation when compared with the LMB-bound cleft of the same CRM1 variant (C^α^ r.m.s.d. of 0.897 Å for cleft residues 509–576; Fig. 3 ▸ *c*) and a further narrowed conformation when compared with the CRM1 cleft occupied by an NES peptide (C^α^ r.m.s.d. of 0.983 Å for cleft residues 509–576; Fig. 3 ▸ *d*). The observed differences in the conformation of the NES-binding cleft indicate that its conformational plasticity allows it to accommodate ligands of different sizes and variable structures. Nevertheless, as presented here, the covalent binding of BME to Cys528 hinders the binding of the ligand of interest, which indicates that BME should be excluded from both the protein-preparation and crystallization processes. Covalent modification of cysteine by BME has been reported in several protein structures [for example, PDB entries 5xhe (Mathur *et al.*, 2018 ▸), 2jpt (Zhukov *et al.*, 2008 ▸) and 1nzu (Nicola *et al.*, 2005 ▸)] and it has been shown to interfere significantly with ligand binding (Mathur *et al.*, 2018 ▸) or to strongly influence protein functionality (Nicola *et al.*, 2005 ▸; Zhukov *et al.*, 2008 ▸).

## 4. Discussion   

Recently, we conceived a crystallization approach that allowed us to gain structural insight into the cysteine-mediated covalent inhibition of human CRM1 by the classical nuclear export inhibitor LMB (Shaikhqasem *et al.*, 2020 ▸). However, when the same method was applied to crystallize a novel inhibitor compound known as C6 (Fetz *et al.*, 2009 ▸), the obtained crystal structure revealed the unexpected covalent modification of Cys528 by BME (Figs. 1 ▸ and 2 ▸), which became an obstacle in obtaining structural information on C6–CRM1 interactions. Although BME was used as a reducing agent during CRM1 purification, the results of mass-spectrometric analysis of the purified protein before and after mixing with crystallization buffer indicate that the modification is induced by the crystallization buffer conditions. The reactivity of both the cysteine and BME is most probably induced due to the higher pH of the crystallization buffer (Poole, 2015 ▸). The utilized crystallization condition (Morpheus condition H10) has a pH of 8.5, which is maintained by a mixture of Bicine and Tris added to 100 m*M*, while the purification buffer has a pH of 7.8 maintained by 50 m*M* HEPES (Table 2 ▸). A pH change can induce reactivity of the cysteine by the deprotonation of its thiol moiety (*R*SH) when increased above its p*K*_a_. The cysteine side chain has a default p*K*_a_ value of 9. However, the p*K*_a_ value can be significantly affected by the microenvironment of the cysteine (Bhatnagar & Bandyopadhyay, 2018 ▸; Klomsiri *et al.*, 2011 ▸). For example, metal-binding enzymatic cysteines were shown to exhibit a lower range of p*K*_a_ values (8.1 ± 2.2) when buried in a hydrophobic cluster (Bhatnagar & Bandyopadhyay, 2018 ▸). Furthermore, BME exhibits decreased stability as the pH increases, which can lead to the formation of covalent adducts with surface cysteines (Wingfield, 1995 ▸). Nevertheless, the covalent adduct of Cys528 and BME was only observed when C6 was used for crystallization and not when LMB was used; the latter was clearly defined in the electron-density map of the crystal structure of the *^Hs^*CRM1^Δ^–RanGTP–LMB complex (Shaikhqasem *et al.*, 2020 ▸). This can be explained by the tight irreversible covalent binding of LMB to Cys528. Surprisingly, crystal structures of LMB bound to human and yeast CRM1 export receptors have revealed that the lactone ring of LMB is hydrolyzed upon binding (Shaikhqasem *et al.*, 2020 ▸; Sun *et al.*, 2013 ▸). Polar interactions with surrounding positively charged residues have been shown to stabilize the lactone ring in an open conformation, which renders the covalent conjunction with LMB irreversible (Sun *et al.*, 2013 ▸). The incubation of CRM1 with an inhibitor prior to crystallization seems to be sufficient for LMB to irreversibly react with the cysteine, as it binds in the nanomolar range (IC_50_ = 151 n*M*; Shaikhqasem *et al.*, 2020 ▸). The resulting stable complex prohibits the possible modification by BME upon subsequent mixing with the crystallization buffer. In contrast, C6 demonstrates weaker binding than LMB (IC_50_ = 4.2 µ*M*; Shaikhqasem *et al.*, 2020 ▸), and due to the possibility of its reversible binding such persistent stability could not be obtained.

Taken together, the presence of BME hinders the binding of the ligand of interest and thereby interferes with the crystallization of CRM1 in complex with novel inhibitor compounds. This interference can be avoided by elimination of BME from the protein-preparation buffer or by screening for a different crystallization condition with a lower pH. Alternatively, undesired modification might be avoided by using reducing agents that demonstrate a higher stability than BME over a wider pH range such as, for example, tris(2-carboxy­ethyl)phosphine (TCEP; Getz *et al.*, 1999 ▸).

## 5. Conclusion   

Here, we have presented the crystal structure of human CRM1 modified at Cys528 by BME, which was used as a buffer component during protein purification. The detected modification hindered the binding of the inhibitor compound, which could not be localized in the electron-density map. In conclusion, our study provides another example of how protein buffer components and conditions can significantly interfere with the characterization of cysteine modifications. Therefore, buffer composition and chemical conditions must be critically considered during protein preparation and further experimentation.

## Supplementary Material

PDB reference: human CRM1 covalently modified by 2-mercaptoethanol, 7b51

## Figures and Tables

**Table 1:** Macromolecule-production information

Macromolecule	*^Hs^*CRM1^Δ^	*^Hs^*Ran^1–180,Q69L^
Source organism	*Homo sapiens*	*Homo sapiens*
Expression vector	pET-21a	pQE80
Expression host	*E. coli* BL21(DE3)	*E. coli* BL21(DE3)pLysS
Complete amino-acid sequence of the construct produced	**MASMTGGQQMGRGS**MPAIMTMLADHAARQLLDFSQKLDINLLDNVVNCLYHGEGAQQRMAQEVLTHLKEHPDAWTRVDTILEFSQNMNTKYYGLQILENVIKTRWKILPRNQCEGIKKYVVGLIIKTSSDPTCVEKEKVYIGKLNMILVQILKQEWPKHWPTFISDIVGASRTSESLCQNNMVILKLLSEEVFDFSSGQITQVKSKHLKDSMCNEFSQIFQLCQFVMENSQNAPLVHATLETLLRFLNWIPLGYIFETKLISTLIYKFLNVPMFRNVSLKCLTEIAGVSVSQYEEQFVTLFTLTMMQLKQMLPLNTNIRLAYSNGKDDEQNFIQNLSLFLCTFLKEHDQLIEKRLNLRETLMEALHYMLLVSEVEETEIFKICLEYWNHLAAELYRESPFSTSASPLLSGSQHFDVPPRRQLYLPMLFKVRLLMVSRMAKPEEAAAVENDQGEVVREFMKDTDSINLYKNMRETLVYLTHLDYVDTERIMTEKLHNQVNGTEWSWKNLNTLCWAIGSISGAMHEEDEKRFLVTVIKDLLGLCEQKRGKDNKAIIASNIMYIVGQYPRFLRAHWKFLKTVVNKLFEFMHETHDGVQDMACDTFIKIAQKCRRHFVQVQVGEVMPFIDEILNNINTIICDLQPQQVHTFYEAVGYMIGAQTDQTVQEHLIEKYMLLPNQVWDSIIQQATKNVDILKDPETVKQLGSILKTNVRACKAVGHPFVIQLGRIYLDMLNVYKCLSENISAAIQANGEMVTKQPLIRSMRTVKRETLKLISGWVSRSNDPQMVAENFVPPLLDAVLIDYQRNVPAAREPEVLSTMAIIVNKLGGHITAEIPQIFDAVFECTLNMINKDFEEYPEHRTNFFLLLQAVNSHCFPAFLAIPPTQFKLVLDSIIWAFKHTMRNVADTGLQILFTLLQNVAQEEAAAQSFYQTYFCDILQHIFSVVTDTSHTAGLTMHASILAYMFNLVEEGKISTSLNPGNPVNNQIFLQEYVANLLKSAFPHLQDAQVKLFVTGLFSLNQDIPAFKEHLRDFLVQIKEFAGEDTSDLFLE**RSRSHHHHHH**	**MG**MAAQGEPQVQFKLVLVGDGGTGKTTFVKRHLTGEFEKKYVATLGVEVHPLVFHTNRGPIKFNVWDTAGLEKFGGLRDGYYIQAQCAIIMFDVTSRVTYKNVPNWHRDLVRVCENIPIVLCGNKVDIKDRKVKAKSIVFHRKKNLQYYDISAKSNYNFEKPFLWLARKLIGDPNLEFVAMP

**Table 2:** Crystallization

Method	Vapor diffusion
Plate type	Sitting drop
Temperature (K)	277.15
Protein concentration (mg ml^−1^)	3
Buffer composition of protein solution	50 m*M* HEPES pH 7.8, 130 m*M* NaCl, 2 m*M* MgCl_2_, 6 m*M* 2-mercaptoethanol
Composition of reservoir solution	10%(*w*/*v*) PEG 8000, 20%(*v*/*v*) ethylene glycol, 100 m*M* Bicine–Tris base pH 8.5, 20 m*M* sodium DL-glutamate, 20 m*M* DL-alanine, 20 m*M* glycine, 20 m*M* DL-lysine–HCl, 20 m*M* DL-serine
Volume and ratio of drop	0.5 µl; 1:1 ratio
Volume of reservoir (µl)	40

**Table 3:** Data collection and processing Values in parentheses are for the outer shell.

Diffraction source	P13, PETRA III, DESY
Wavelength (Å)	0.9762
Temperature (K)	100
Detector	PILATUS 6M
Crystal-to-detector distance (mm)	576.1
Rotation range per image (°)	0.1
Total rotation range (°)	222
Exposure time per image (s)	0.05
Space group	*I*222
*a*, *b*, *c* (Å)	121.11, 150.59, 231.97
α, β, γ (°)	90, 90, 90
Resolution range (Å)	126.31–2.58 (2.74–2.58)
Total No. of reflections	519292 (48302)
No. of unique reflections	65209 (9016)
Completeness (%)	97.5 (84.3)
Multiplicity	7.96 (5.36)
〈*I*/σ(*I*)〉	16.60 (1.95)
*R*_meas_	0.083 (0.712)
CC_1/2_ (%)	99.9 (81.7)
Overall *B* factor from Wilson plot (Å^2^)	64.7

**Table 4:** Structure solution and refinement Values in parentheses are for the outer shell.

Resolution range (Å)	126.31–2.58 (2.65–2.58)
Completeness (%)	97.5
σ Cutoff	*F* > 0.0σ(*F*)
No. of reflections, working set	61972 (3242)
No. of reflections, test set	3237 (149)
Final *R*_cryst_	0.214 (0.544)
Final *R*_free_	0.250 (0.583)
No. of non-H atoms	
Protein	9613
Ligand	37
Water	136
Total	9786
R.m.s. deviations	
Bonds (Å)	0.008
Angles (°)	1.605
Average *B* factors (Å^2^)	
Protein	62.2
Ligand	50.2
Water	59.9
Ramachandran plot	
Most favored (%)	97.0
Allowed (%)	3.0
PDB code	7b51
